# The influence of the starvation–predation trade‐off on the relationship between ambient temperature and body size among endotherms

**DOI:** 10.1111/jbi.12695

**Published:** 2015-12-22

**Authors:** John M. McNamara, Andrew D. Higginson, Simon Verhulst

**Affiliations:** ^1^ School of MathematicsUniversity of BristolBristolBS8 1TWUK; ^2^ Centre for Research in Animal BehaviourCollege of Life and Environmental SciencesUniversity of ExeterExeterEX4 4QGUK; ^3^ School of Biological SciencesUniversity of BristolBristolBS8 1TQUK; ^4^ Groningen Institute for Evolutionary Life SciencesUniversity of Groningen9747 AGGroningenThe Netherlands

**Keywords:** Bergmann's rule, body composition, energy balance, fat storage, food shortage, heat conservation, latitude, NPP, resource availability, starvation resistance

## Abstract

**Aim:**

The tendency for animals at higher latitudes to be larger (Bergmann's rule) is generally explained by recourse to latitudinal effects on ambient temperature and the food supply, but these receive only mixed support and do not explain observations of the inverse to Bergmann's rule. Our aim was to better understand how ecological variables might influence body size and thereby explain this mixed support.

**Location:**

World‐wide.

**Methods:**

Previous explanations do not allow for the selective pressure exerted by the trade‐off between predation and starvation, which we incorporate in a model of optimal body size and energy storage of a generalized homeotherm. In contrast to existing arguments, we concentrate on survival over winter when the food supply is poor and can be interrupted for short periods.

**Results:**

We use our model to assess the logical validity of the heat conservation hypothesis and show that it must allow for the roles of both food availability and predation risk. We find that whether the effect of temperature on body size is positive or negative depends on temperature range, predator density, and the likelihood of long interruptions to foraging. Furthermore, changing day length explains differing effects of altitude and latitude on body size, leading to opposite predictions for nocturnal and diurnal endotherms. Food availability and ambient temperature can have counteracting selective pressures on body mass, and can lead to a non‐monotonic relationship between latitude and size, as observed in several studies.

**Main conclusions:**

Our work provides a theoretical framework for understanding the relationships between the costs and benefits of large body size and eco‐geographical patterns among endotherms world‐wide.

## Introduction

The tendency for body size to increase with latitude was observed over 150 years ago (Bergmann, 1847), and is known as Bergmann's rule. Bergmann's rule is one of the best studied macro‐ecological relationships, and meta‐analyses of within‐species studies of birds (Ashton, 2002) and mammals (Clauss *et al*., 2013) have found that Bergmann's rule holds for endotherms throughout the world. However, the causal mechanism has still not been identified, and formal modelling to derive comprehensive explanations of the observed patterns is scant. The original proposal relates to the need to conserve heat, because colder ambient temperatures might select for animals with lower surface‐area‐to‐volume ratios, which declines as body size increases (Bergmann, 1847; Peters, 1983). However, this does not necessarily mean that an animal should be as large as possible in cold conditions because the absolute rate of heat loss increases with size, meaning greater energy requirements (McNab, 1971; Ergon *et al*., 2004). Greater energy requirements makes starvation more likely, and more time must be spent foraging for food, perhaps exposing the animal to its predators. In order to understand how the selective pressure to avoid death from heat loss will have influenced body size and composition we must also consider the energetic trade‐off between the risks of starvation and predation (Lima, 1986; McNamara & Houston, 1987; Cresswell *et al*., 2009; Bennett *et al*., 2013).

The heat‐conservation hypothesis is consistent with the general lack of adherence to Bergmann's rule among ectotherms (arthropods, fish, reptiles: Pincheira‐Donoso, 2010; amphibians: Adams & Church, 2008), which do not need to maintain their temperature to stay alive. By contrast, the powerful impact of thermoregulation requirements for endotherms are indicated by the seasonal changes of size in winter among several mammals not attributable to changes in the food supply (Churchfield *et al*., 2012) and in response to a warming climate (Gardner *et al*., 2011). The heat conservation hypothesis suggests that Bergmann's rule should hold more for temperate than tropical taxa because the latitudinal trend in temperature is less steep in the tropics (McNab, 1971) and indeed the relationship between latitude and size varies over latitude as predicted (McNab, 1971; Meiri & Dayan, 2003; Rodriguez *et al*., 2008). Several other studies with more detailed mapping of ambient temperature have suggested that temperature rather than other correlates of latitude is the most likely causal factor for Bergmann's rule among birds (Olson *et al*., 2009) and mammals (Freckleton *et al*., 2003). However, several studies have found a non‐monotonic relationship; with body size increasing with decreasing temperature at low temperatures but decreasing with decreasing temperature at high temperatures (Blackburn & Hawkins, 2004; Rodriguez *et al*., 2008; Morales‐Castilla *et al*., 2012). For instance, in the tropics body size gets smaller as altitude increases and so temperature decreases (Rodriguez *et al*., 2008). Some taxa (e.g. shrews, Ochocinska & Taylor, 2003; birds, Morales‐Castilla *et al*., 2012) show negative, positive, and no associations between body size and temperature among species in the same geographical regions. Thus, the heat conservation hypothesis is not only lacking in its consideration of the effect of body size on energy requirements and predation risk, but it also lacks explanatory power with respect to qualitative deviations from Bergmann's rule.

Explanation for these deviations from the general trend may depend on the assumptions underlying the heat conservation hypothesis, and that other ecological parameters vary with latitude. Indeed, one possible alternative explanation for Bergmann's rule is environmental productivity (e.g. ecologically relevant net primary production, eNPP), which influences the rate of energy gain and thereby may limit body size (Rosenzweig, 1968; Geist, 1987; McNab, 2010; Huston & Wolverton, 2011). These explanations are based on ideas of food availability during summer, but the characteristics of the food supply in winter may be more important, and shortage of food in winter may select for smaller body sizes, as smaller bodies require less food (Ochocinska & Taylor, 2003). Rodriguez *et al*. (2008) suggested that fasting endurance is a more important factor than heat conservation, and larger animals catabolize energy reserves at lower rates per unit of mass and so should be favoured in seasonal and unpredictable environments.

Authors have seemed to assume that explanations are mutually exclusive, with researchers attempting to partition out the relative magnitudes of effects, and with only rare instances of acknowledgement that several pressures work in concert (e.g. Rodriguez *et al*., 2008; McNab, 2010; Yom‐Tov & Geffen, 2011; Pincheira‐Donoso & Meiri, 2013). This partitioning approach results in different weightings ascribed to each explanation depending on location and taxon. In our view, this partitioning approach pervades this research because we are lacking an overarching functional model that integrates ecological determinants of body size. Furthermore, resistance to starvation does not depend on body size *per se*, but on the ratio of energetic reserves to energetic requirements. Most energy reserves are in the form of fat yet fat is energetically inexpensive to maintain compared to muscle and organs (Glazier, 2005), and so effects of body size are complicated if the proportion of body size accounted for by individual components of the body (e.g. fat) vary among populations or species. Any attempt to understand the dependence of body size on temperature must consider how body size and composition (e.g. fat stores) affect performance measures such as ability to get food and avoid predators and consider interactions among temperature, food availability and the risks of starvation and predation. We developed such a model for a generalized homeotherm, supposing that both temperature and resource availability play a role in determining overwinter survival, when death from starvation mostly occurs. We use this model to expose the logic of the heat conservation hypothesis, and show how several ecological variables may interact to generate the range of observed relationships between latitude and animal body size across the world.

## The model

We consider a homoeothermic animal that has evolved to survive overwinter (when it is not growing or reproducing), where the two causes of mortality are starvation and predation. We assume that conditions in the winter – such as ambient temperature, food availability and the risk of predation – are constant, except that there are occasional interruptions to the food supply. We are interested in assessing how sizes of the body components are optimized – minimizing total mortality rate – to the environmental conditions.

### Body components

In our model, there are three components to the animal's body. The first, the feeding apparatus (e.g. beak) plus supporting muscle is fixed at a given size (*F*). This allows us to avoid the complication of allowing the type of prey that can be ingested to depend on body size and so the need to specify the size distribution of prey in the environment. We also ignore the fact that the capacity to process food may need to increase as the amount of food ingested increases. Our assumptions are motivated by considering the optimal design of the body given a particular foraging niche, and in this way, we adopt the broadly accepted view that Bergmann's rule only applies among closely related species and between populations of species (Meiri *et al*., 2007; Morales‐Castilla *et al*., 2012).

The other two body components are optimized to the environment. Lean body mass (*L*) includes the mass of those muscles used in locomotion and any elements such as the skeleton that we assume to scale isometrically with the amount of muscle (Prange *et al*., 1979). The third component is the mass of energy reserves (*R*), which are used to maintain metabolism when there is no food, but may reduce the ability to evade predators or to catch prey. For simplicity, *R* is a quantity of both mass of fat reserves and the energy it contains, assuming an implicit conversion. Total body mass is *M* = *F* + *L* + *R*.

We assume that the tissue‐specific metabolic rates of the components are proportional to their mass: i.e. a unit increase in each component increases metabolic costs by a constant amount. This assumption is reasonable because we are concerned with relationships within species or between closely related species, and within‐species rates are roughly constant (Peters, 1983). This constant amount can, however, differ between components. Basal metabolic rate *c*_0_ is taken to be of the form

(1) $$c_{0}=m_{F}F+m_{L}L+m_{R}R$$

where *m*_*F*_ *, m*_*L*_ and *m*_*R*_ are constants specific to each component. Basal metabolic rate is defined as the energy consumption when the animal is inactive (i.e. usually measured at night in diurnal animals), not digesting, and not doing thermogenesis.

### Critical temperatures

The resting metabolic rate *c*_rest_ depends on body composition and ambient temperature, as the animal may have to use energy in thermoregulation. We assume that the minimum heat loss while resting is *kM* ^σ^(*T*_b_–*T*), where *T*_b_ is body temperature, *T* is ambient temperature, σ controls how heat loss increases with mass, and *k* is a constant energy per unit of effective surface per temperature difference (because *M* ^σ^ is proportional to the effective surface area) that is determined by the animal's thermal insulation properties. Over a large size range metabolic rates of mammals do not follow a simple power law (Kolokotrones *et al*., 2010), but for species within a given foraging niche the deviation from a linear relationship (on a log‐log scale) is small, and therefore our assumption of a simple power law is reasonable. Numerical results are based on σ = 2/3, as expected from Euclidean arguments about the scaling between surface area and volume (Peters, 1983). However, from models that allow for non‐uniform temperatures across an insulated furry body the value of σ is predicted to be 0.5 (Porter & Kearney, 2009). Thus, our analytic results assume only that σ lies between zero and unity, and apply generally for values of σ in this range.

We define the critical temperature *T*_rest_ by *kM* ^σ^(*T*_*b*_–*T*_rest_) =* c*_0_, where the baseline metabolism is exactly sufficient to keep the animal warm, i.e. there are no additional thermoregulation costs. As we are concerned with surviving cold we do not consider the case when the temperature is above the thermo‐neutral zone of the animal (a valid assumption for an endotherm in winter or during the night). Thus, we ignore costs incurred in losing excess heat and assume that the animals pays the cost of keeping warm or the baseline cost, whichever is the larger. Formally, resting metabolic rate is

(2) $$c_{\mathrm{rest}}=\left\{\begin{array}{cc} c_{0} & \text{if}\;T\geq T_{\mathrm{rest}}\\ kM^{\sigma }\left(T_{b}-T\right) & \text{if}\;T<T_{\mathrm{rest}} \end{array}\right.$$

We assume that activity requires an additional energy expenditure of *aM* where *a* is a dimensionless constant. Of this, a proportion *p* creates internal heat that helps keep the animal warm (‘energy substitution’, sensu McNamara *et al*., 2004). Then, we can identify a second critical temperature *T*_active_ as the temperature above which an active animal is thermo‐neutral. This temperature satisfies *kM* ^σ^(*T*_*b*_–*T*_active_) =* c*_0_ + *paM*. Below this critical temperature the heat generated by activity is not sufficient to maintain body temperature, so that even an active animal must generate extra heat by shivering or non‐shivering thermogenesis. Thus, the metabolic rate when active is

(3) $$c_{\mathrm{active}}=\left\{\begin{array}{cc} c_{0}+aM & \text{if}\;T\geq T_{\mathrm{active}}\\ c_{\mathrm{rest}}+\left(1-p\right)aM & \text{if}\;T<T_{\mathrm{active}} \end{array}\right.$$

When food is available the animal maintains its level of energy reserves at *R* by foraging for a proportion ρ of each 24 h period. ρ is calculated from the energy balance equation that results from the animal having a stable body composition when food is available:

(4) $$\rho \gamma =\left(1-\rho \right)c_{\mathrm{rest}}+\rho c_{\mathrm{active}}$$

where γ is the rate of energy gain when foraging, measured in units of mass of fat gained per day of foraging. Manipulating the energy balance equation (4) gives:

(5) $$\rho =\frac{c_{\mathrm{rest}}}{\gamma +c_{\mathrm{rest}}-c_{\mathrm{active}}}$$

Note that ρ is influenced by the choice of *L* and *R*, and ρ in turn influences the animal's energy costs, rate of food consumption, and exposure to predators. As an animal may be restricted to foraging only during daylight (or darkness), we assume that ρ is restricted to lie in the range ρ ≤ ρ_max_, where ρ_max_ is the available proportion of each 24 h period during which foraging is possible.

### Environmental parameters

Foraging is sometimes not possible (due to e.g. bad weather), whereupon animals have to rely on their energy reserves. We assume that foraging interruptions occur with rate ϕ. When an interruption occurs an animal must rest and will die from starvation if its energy reserves are exhausted. We ignore the possibility that resting metabolic rate may change as fat is lost and *M* declines, as in non‐specialized species fat is a poor insulator (Pond, 1992). For simplicity we ignore catabolism of muscle. If the animal starts the period with energy reserves *R* it can survive for time

(6) $$\tau =\frac{R}{c_{\mathrm{rest}}}$$

Increasing lean body size *L* increases *c*_rest_ and hence decreases starvation resistance. The probability that an interruption lasts longer than time *t* is *S*(*t*) and so the probability the animal starves during an interruption is *S*(τ). We assume that these interruptions are rare and spaced out, so that if an animal survives an interruption its reserves return to the optimal level (R*) before the next interruption.

Under the above assumptions the rate of starvation – the mortality from starvation per unit time – is ϕ*S*(τ). Assuming that the animal is only at risk of predation while foraging, the rate of predator attack while foraging is α, and the probability that the animal is killed if a predator attacks is β, then the rate of mortality from predation is ραβ. Thus, the total rate of mortality, μ, is given by

(7) μ=ϕS(τ)+ραβ

We are concerned with the value of lean body mass L* and energy reserves R* that minimizes μ and so maximizes survival over any time period.

Baseline values of parameters used to produce the figures are given in Table 1. To aid intuition, one could consider the animal to be a small bird weighing around 45 grams and a unit of time to be 1 day (24 h). Birds have body temperatures (*T*_b_) around 40 °C (Clarke & Rothery, 2008) and heat loss is likely to scale with the surface area to volume ratio (so σ = 2/3). We assume that a lean bird uses energy at 3× basal metabolic rate when foraging (Alexander, 2005). For the baseline values, an optimal bird would be 20–30% fat, which is realistic (A. D. Higginson & J. Wells, unpublished data). The metabolic cost values (*m*_*F*_ *, m*_*R*_ *, m*_*L*_) are such that this optimal bird uses around 5 g of fat per day to meet its energetic needs. The bird is attacked by a predator once every 10 days (α = 0.1). An interruption to the food supply happens once every 10 days (ϕ = 0.1) but only a one‐in‐26 interruptions last longer than a single day [*S*(τ *=* 1)]. Other parameters (*a*, γ) were set such that an optimal bird has 50–70% chance of surviving a winter of 100 days, to fit with observations of passerines in Northern Europe (Gullett *et al*., 2014). The model could apply to any endothermic animals. For instance, for a large mammal mass units could be thought of as kilograms, and the unit of time as 10 days.

**Table 1:** Parameters in the model and their default values

Symbol	Description	Default value
*F*	Mass of feeding apparatus	4
*L*	Mass of lean tissue (muscle, skeleton, organs)	–
*R*	Mass of energy reserves (mostly adipose tissue)	–
*M*	Total body mass	–
*m*_*F*_	Metabolic cost of feeding apparatus/unit mass	0.125
*m*_*R*_	Metabolic cost of fat/unit mass	0.05
*m*_*L*_	Metabolic cost of muscle/unit mass	0.125
*T*_b_	Body temperature	40
*k*	Proportionality constant in heat loss	0.01575
σ	Scalar of size to heat loss	2/3
*a*	Activity cost per unit mass	0.25
*p*	Proportion of generated energy for energy substitution	0.25
γ	Gross rate of energy gain	40
α	Predator attack rate	0.1
ϕ	Rate of interruptions to the food supply	0.1
β	Vulnerability to predators	$\frac{1}{1+100{\left(\frac{L}{M}\right)}^{4}}$
*S*(τ)	Probability that interruption lasts longer than τ	$\frac{1}{1+25\tau^{3}}$

## Results

Firstly, we consider the case where both predation susceptibility β and intake rate γ are constant: not affected by muscle mass *L* or energy reserves *R*. Then for given *R* both the active and resting metabolic rates increase with increasing *L*, so that the proportion of time spent foraging (ρ) increases with increasing *L*. Also, the time that can be survived without food (τ) decreases as *L* increases. As both predation and starvation rates increase with *L* it is optimal to have as little muscle as possible. This result holds independent of temperature.

In order to assess the dependence of body composition on temperature in more realistic situations, we will assume in the following that either the rate of energy gain (γ) or the susceptibility to predation (β) is affected by *L* and *R*. γ may change with body composition if being more muscular means the animal can better compete with conspecifics or capture prey. β may increase with the ratio *R* to *L* if fat load impairs the evasion of predators. We initially keep γ as a constant and assume that β is a decreasing function of *L/M*, but the insights we achieve are unchanged if instead we assume that γ increases with *L/M* and β is constant. Note that we assume that the rate at which predators attack while foraging (α) is not affected by the total body size or the size of components.

### Discontinuities at the critical temperatures

**Figure 1:**
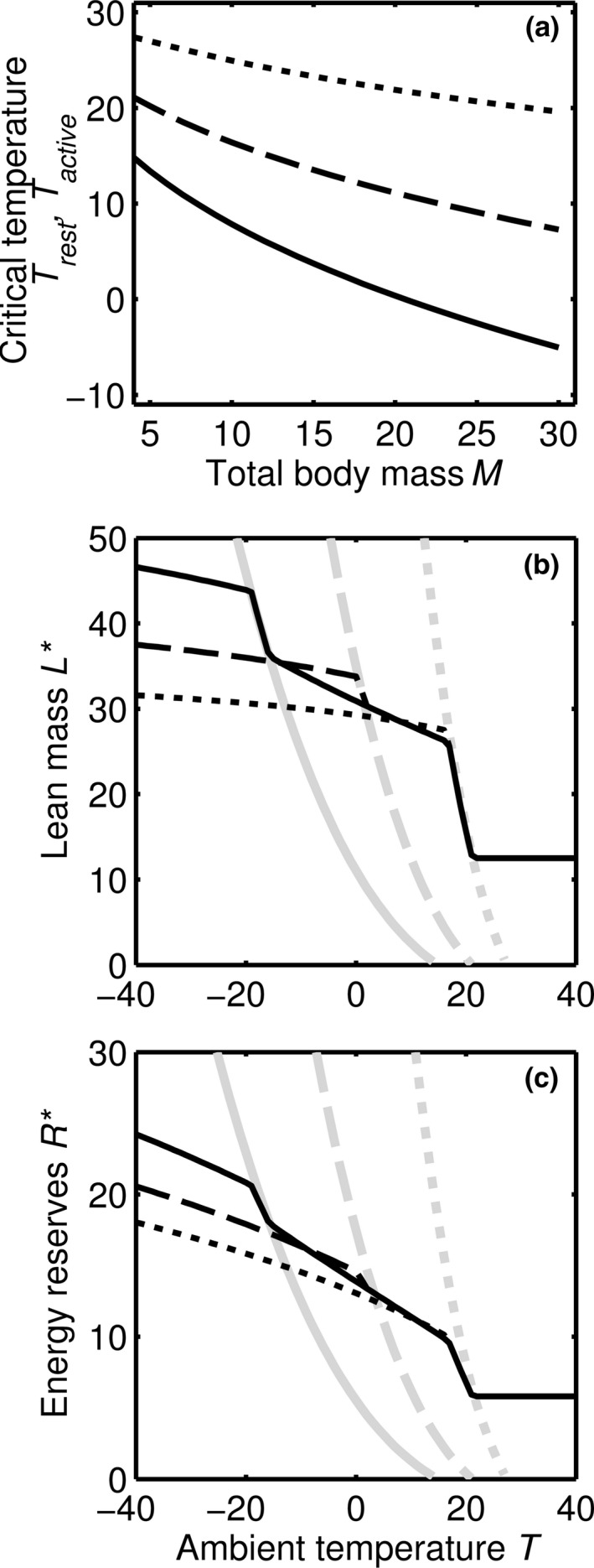
The importance of critical temperatures. (a) The relationship between total body mass *M* and the temperatures at which the animal must initiate thermogenesis *T*_active_ and *T*_rest_. The effect of temperature on (b) optimal lean mass L* and (c) optimal energy reserves R*. In all panels we assume *F = *4, and show results for three values of energy substitutability *p*: no substitution (*p *=* *0, dotted lines), moderate energy substitution (*p *=* *0.25, dashed lines) and high energy substitution (*p *=* *0.5, solid lines). Note that if *p *=* *0 then *T*_rest_ *= T*_active_. In (b) and (c) we also show the relationship between temperature and the critical component masses below which the animal must generate heat by thermogenesis *M*_active_ (grey lines) for the same three values of *p* (patterns as above), assuming *L *=* *2*R*. Note that the optimal strategy tracks *M*_active_ at temperatures where the strategy changes quickly as *T* changes. Other parameters as in Table 1.

The critical temperature above which thermogenesis is not necessary when resting (*T*_rest_) decreases with increasing *M* (Fig. 1a), due to the lower surface to volume ratio with increasing mass. For a given ambient temperature *T* there is a critical total body mass *M*_rest_ (*T*) such that the animal is below thermoneutrality for *M < M*_rest_(*T*) and is thermoneutral for *M ≥ M*_rest_(*T*). In the thermoneutral zone, resting energy use increases linearly with *L* and *R*. Below this zone, energy use scales with *M* ^σ^. Thus, the rate of increase in resting energy expenditure with respect to *M* increases by a factor of 1/σ as *M* increases from below *M*_rest_(*T*) to above *M*_rest_(*T*) (see Appendix S1 in Supporting Information). In particular, when σ = 2/3 the marginal increase in resting energy expenditure with increasing *M* increases by 50% at *M*_rest_(*T*). Often optimal body mass M* is as large as possible without incurring this extra marginal cost [M* = M_rest_(*T*)]; body mass is large enough to avoid the need for thermogenesis but no larger. The optimal strategy thereby trades off the starvation rate associated with increased costs of keeping warm against the mortality rate during foraging. *M*_rest_(*T*) increases as *T* decreases and so for some temperature ranges M* rapidly increases as temperature decreases, as M* tracks *M*_rest_(*T*). Thus, we predict the general relationship between temperature and size as in Bergmann's rule.

When energy spent on activity can help keep an animal warm (substitution *p *>* *0) there is a second critical temperature *T*_active_. Below this temperature even the energy generated by foraging activity is insufficient to keep the animal warm. As the amount of heat produced by basic processes and activity depend on body mass, for a given temperature *T* there is a critical body mass *M*_active_(*T*), above which an active animal is large enough to avoid thermogenesis. *M*_active_(*T*) is smaller for larger values of *p*. Therefore, at a temperature that depends on *p* there is a second sudden increase in M* where M* tracks *M*_active_(*T*) over some temperature range. These effects are shown for a particular form for *S*(τ) and for our baseline parameter values in Figure 1(b,c). The above depends on the mass of the feeding apparatus being non‐negligible (*F *>* *0). Computations suggest that L* always increases with increasing *F* until the maximum rate of foraging (ρ_max_) is reached. The values of the critical temperatures depend on *F* and so *F* will affect how the other body components respond to temperature, most obviously shifting the position of the ‘steps’ (see Fig. S1 in Appendix S2).

### Effect of the distribution of interruption times under cold conditions

**Figure 2:**
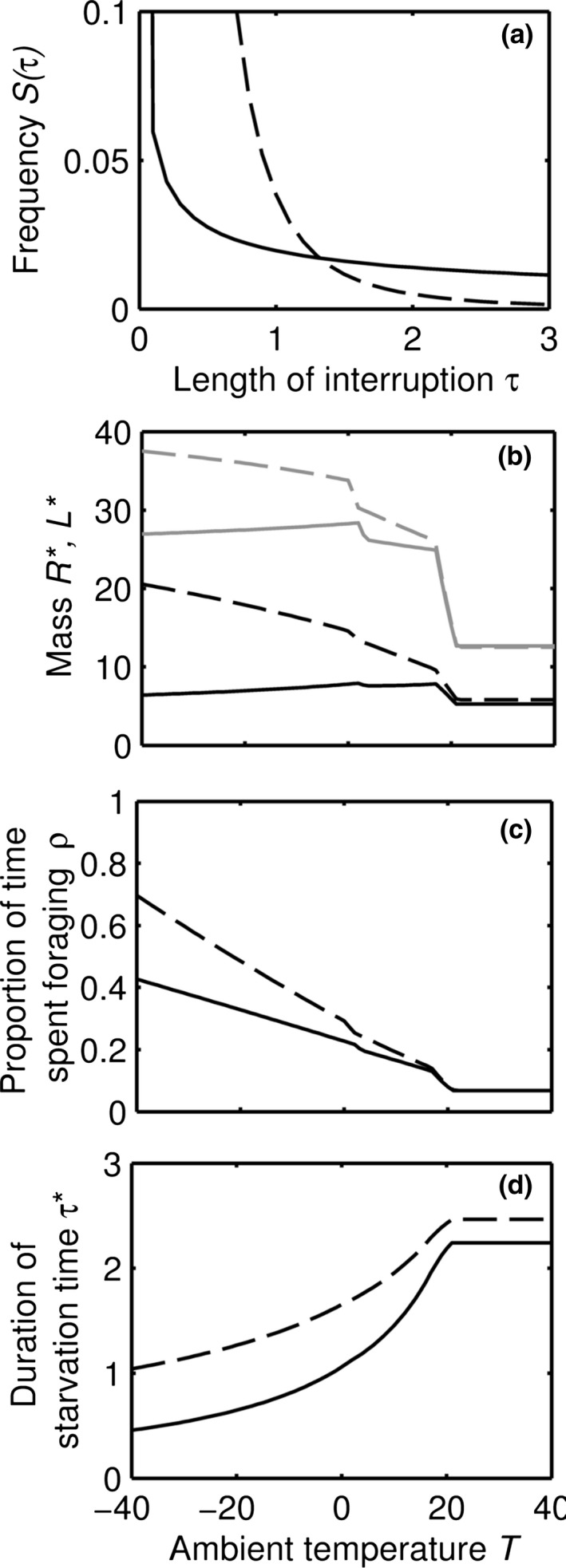
Effect of the shape of the function *S* specifying the length of interruptions. (a) We compare $S\left(\tau \right)=\frac{1}{1+25\tau^{3}}$ (dashed lines) which is short‐tailed (see text) for almost all its range and $S\left(\tau \right)=\frac{1}{1+50\tau^{05}}$ (solid lines) which is long‐tailed for almost all its range. In each case results presented are for the optimal body composition as a function of ambient temperature: (b) Optimal lean mass L* (gray) and energy reserves R* (black); (c) the optimal proportion of time spent foraging (ρ); and (d) the time that an animal can survive (τ*). Other parameters as in Table 1.

We explored effects of the distribution of interruption times by altering the function *S*(τ). If the temperature is so low that the heat generated by activity is not sufficient to keep the animal warm (*T < T*_active_), then whether M* decreases with temperature depends upon the shape of the relationship between *S* and τ (Fig. 2). We find in Appendix S3 that whether M* increases or decreases with temperature below *T*_active_ depends on the sign of the value

(8) $$2S^{\prime }\left(\tau^{*}\right)+\tau^{*}S^{\prime \prime }\left(\tau^{*}\right)$$

As the risk of starvation will always decline with increasing energy reserves the first term is negative. The second term may be positive or negative. If the second term is positive the sign of equation (8) depends on the relative magnitudes of the two terms at τ*. As the rate of change in *S*(τ) with respect to τ determines the size of the tail at extreme values, we refer to distributions for which 2*S* ^′^(τ*) + τ*S ^′′^(τ*) is positive (in the neighbourhood of τ*) as short‐tailed and distributions for which 2*S* ^′^(τ*) + τ*S ^′′^(τ*) is negative as long‐tailed. Examples of functions that are short‐tailed and long‐tailed for almost all their range are shown in Figure 2(a). In both cases both muscle and energy reserves increase as temperature decreases below *T*_rest_, with a second ‘step’ increase as the temperature falls below *T*_active_ (Fig. 2b). When *S*(τ) is short‐tailed, both muscle and energy reserves increase even more below *T*_active_ as temperature decreases further. This occurs because it pays to carry enough energy reserves to survive long famines, and L* increases so as to keep the predation rate below the value of *L/M* associated with a steep increase in β (see Fig. S2 in Appendix S2).

In contrast, both muscle and energy reserves decrease below *T*_active_ as temperature decreases when the distribution is long‐tailed. This occurs because increasing investment in reserves increases the number of famines that will be survived at a less dramatic rate, and so the animal should instead reduce costs by reducing L* and so have to reduce R* to limit the increase in the predation rate. In Appendix S3 we show that the effect of the sign of equation (8) on the slope of M* holds generally. The optimal proportion of time spent active (ρ*) increases as the ambient temperature gets colder because the animal must gather more energy to survive. This increase is much greater when interruptions are short‐tailed (Fig. 2c) because the animal should also get larger as temperature decreases (Fig. 2b), which further increases energetic requirements. As the temperature decreases, the optimal length of time until starvation (τ*) declines because the metabolic rate increases (Fig. 2d). In conclusion, the distribution of interruption times in our model [*S*(τ)] critically determines whether optimal body size increases with decreasing temperature (Bergmann's rule) or decreases with decreasing temperature (opposite to Bergmann's rule).

### Effect of environmental parameters

Increasing the frequency of interruptions to foraging (ϕ) leads to an increase in R* (see Fig. S3 in Appendix S2). When the temperature is cold (e.g. −30 °C), this is accompanied by an increase in L*. As the frequency of predator attack (α) increases L* and R* decrease as it is better to reduce exposure than vulnerability, whereas increasing food availability (γ) causes an in increase in both L* and R*. These latter effects are robust and do not depend on other model details. Note that ϕ has a very slight effect on M* unless γ is large. Despite this, the magnitude of ϕ can qualitatively affect the relationship between temperature and optimal body size, as we now demonstrate.

If interruptions end with a constant probability, the duration of interruptions will follow an exponential distribution [*S*(*t*)=e^−*Kt*^], and expression (8) becomes

(9) $$-2Ke^{-K\tau^{*}}+\tau^{*}K^{2}e^{-K\tau^{*}}$$

**Figure 3:**
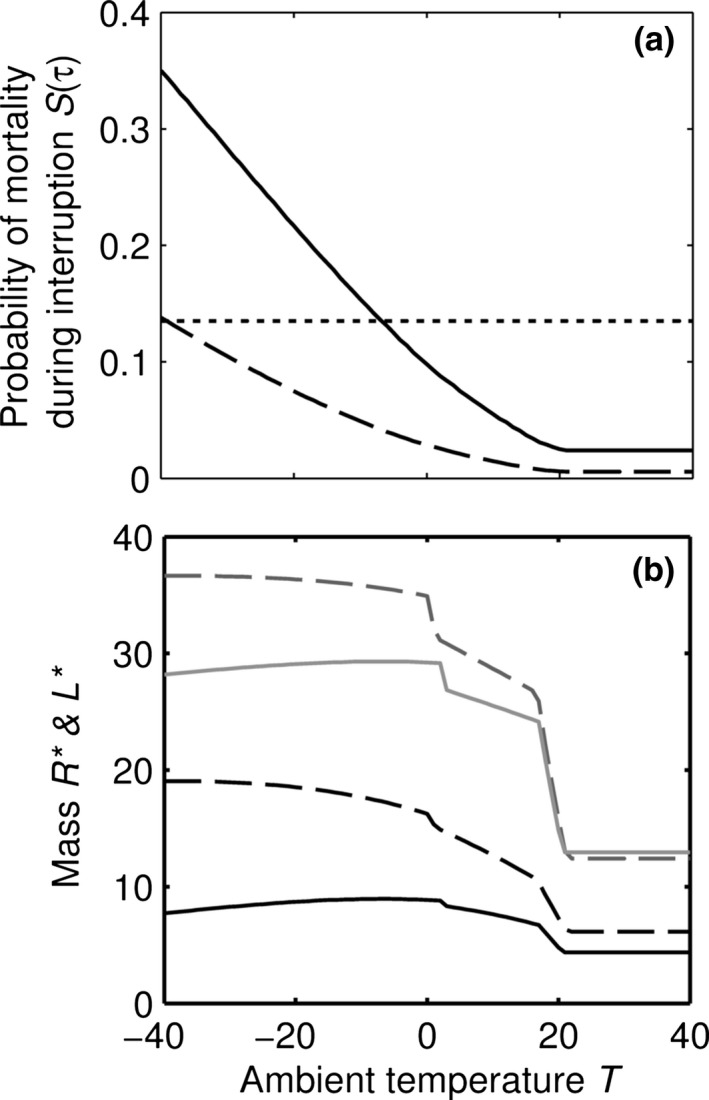
Effect of changing the interruption rate and predator attack rate when the length of interruptions are exponentially distributed (S(τ) = e^−2τ^). The two cases illustrated are (solid lines) ϕ = 0.02, α = 0.4, (dashed lines) ϕ = 0.08, α = 0.125. (a) The probability of mortality during an interruption at the optimal size of body components. The region of the exponential curve is short‐tailed if *S*(τ*) < 0.135 (dotted line) and is long‐tailed if *S*(τ*) > 0.135, see text. (b) Optimal lean mass L* (gray) and energy reserves R* (black) for the two cases. Other parameters as in Table 1.

Expression (9) is negative [indicating *S*(τ) is long‐tailed in this region] when $\tau^{*}<\frac{2}{K}$ and positive [indicating *S*(τ) is short‐tailed in this region] when $\tau^{*}>\frac{2}{K}$. The borderline value $\tau^{*}=\frac{2}{K}$ is such that an animal that can survive time τ* without food has a probability *e* ^−2^ = 0.135 of starving when an interruption occurs. In Figure 3 we consider two cases for which the total mortality rate is similar to the baseline case. In the first case, ϕ is small and α is high. In this case R* is such that $\tau^{*}<\frac{2}{K}$ and *S*(τ*) < 0.135 (Fig. 3a), and below *T*_active_ both L* and R* decrease as temperature decreases (Fig. 3b). In the second case ϕ is larger and α is smaller than in the first case. We now have $\tau^{*}>\frac{2}{K}$ and *S*(τ*) > 0.135, and below *T*_active_ both L* and R* increase as temperature decreases. Thus, the relative magnitude of the risks of starvation and predation determine whether the exponential distribution influenced the optimal strategy as though it were short‐ or long‐tailed. This occurs because when interruptions are rare it pays less to be prepared for them, and so the animal should give more weight to reducing predation. Thus, we have identified two more ecological variables that can alter the sign of the relationship between temperature and body size.

### Susceptibility to predation

Next, we were interested in how the function controlling susceptibility to predation (β) affects the optimal strategy. Interestingly, we find that the form of β (see Fig. S2 in Appendix S2) qualitatively interacts with the parameters of *S*(τ) in determining whether mass should increase or decrease as temperature gets colder below *T*_active_ (see Appendix S3). If predation decreases exponentially with the proportion of the body that is lean mass (*L/M*) then M* decreases as *T* decreases below *T*_active_ when interruptions are long tailed, but when interruptions are short‐tailed M* increases as the temperature decreases below *T*_active_. This is the same as we observed above for a sigmoid curve (Fig. 2). By contrast, if predation decreases linearly as *L/M* increases, long‐tailed interruptions lead to M* increasing as temperature decreases and short‐tailed interruptions lead to M* decreasing as temperature decreases. Thus, the form of the dependence of predation risk on the size of body components can also determine whether we predict adherence to Bergmann's rule or its inverse.

### Effect of the correlations among environmental variables

Several environmental variables change with latitude (Blackburn & Hawkins, 2004) and so these variables will correlate with temperature. Most obviously, day length in winter is shorter at higher latitude. Responsiveness to changes in temperature may be constrained by the amount of time available for foraging (ρ_max_). If temperature gets sufficiently low that the optimal ρ* would ideally be greater than ρ_max_ then the optimal strategy is for M* to decline with further decreases in temperature (see Fig. S4 in Appendix S2) as the animal must decrease the energy demands of a larger body in order to be able to afford the greater costs of staying warm. Thus, we predict that body size in diurnal endotherms will decline with decreasing temperature when days are very short – i.e. close to the poles – but not at high altitude where temperature and day length are not correlated. Nocturnal endotherms, on the other hand, will be able to increase body size as their foraging time is less limited in winter.

**Figure 4:**
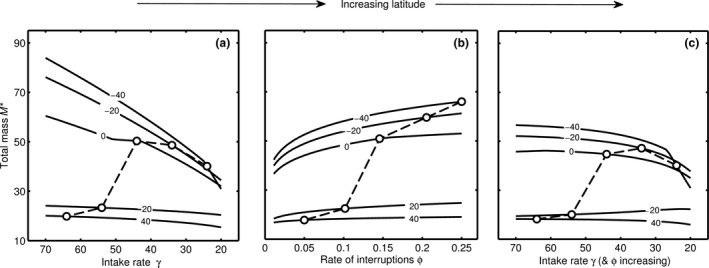
Effect on total body mass *M* of (a) decreasing intake rate γ, (b) increasing rate of interruptions ϕ, (c) decreasing intake rate γ and increasing rate of interruptions ϕ for five values of the ambient temperature (shown on lines). Dashed lines indicate hypothetical conditions along a latitudinal gradient as temperature is correlated with γ and ϕ.

In Figure 4 we explore the impact on M* of decreasing intake rate (γ) and increasing rate of interruptions (ϕ) and both simultaneously for various values of ambient temperature. M* increases with γ (Fig. 4a) and ϕ (Fig. 4b) and can increase or decrease when both change together depending on temperature (Fig. 4c). Since these parameters may both change with temperature, we can connect points across the lines which correspond to changing latitude. In doing so, we see that we can predict non‐monotonic relationships between latitude and body size.

## Discussion

Bergmann's rule has been well‐studied and often but not always confirmed, with some taxa even showing the opposite pattern. A theoretical framework explaining this diversity of patterns by recourse to the selective pressures has been lacking, leaving this variation as well as Bergmann's original rule unexplained from an adaptive viewpoint. We developed a simple optimization model of survival in an endotherm and shown that optimal body size can both increase or decrease with temperature, thereby providing a possible explanation for the mixed agreement with Bergmann's rule. Whether Bergmann's rule will be observed in a taxon should depend on several important aspects of the animal's physiology and environment, including the incidence of foraging interruptions (ϕ), relative likelihood of long food shortages [S(τ)], predator density (α), and predator evasion strategy (influencing β).

The ecological variables that correlate with latitude (or altitude) will all influence the optimal body size, and apparently stronger relationships between one of them in a correlative study cannot imply that one cause of Bergmann's rule is any more ‘correct’ than any other. For instance, it is the dramatic effect of ϕ on the slope of the relationship between temperature and body size that implies that resistance to starvation and heat conservation are mutually interacting determinants of body size. Hence, it may be meaningless to debate the weightings of the different factors, and indeed different weightings are more likely to reflect variance of these ecological parameters in the particular study. Appreciation of this complexity enables us to understand different trends at different temperatures and non‐monotonic relationships between latitude and body size among and within taxa. Our ranges of these parameters we explore are of course arbitrary, and it is likely that many different qualitative relationships can be predicted, depending on local conditions. Thus, we might not expect consistency across the animal kingdom and geographical regions, even though thermoregulation has a strong influence on body mass in all conditions.

Studies inevitably sample individuals across a range of temperatures. A clear outcome of our analysis is that we expect the slope of body size on temperature to be highly variable across the range, with large steps at critical values. These sudden steps – the existence of which could be empirically verified – are caused by tracking the critical body mass at some temperatures, and mean that the magnitude of the relationship between temperature and body size is sensitive to the temperature range studied. Thus, the slope of this relationship observed in studies will depend on the temperature range. This may explain why even closely related species show inconsistency in adherence to Bergmann's rule. These critical temperatures are also affected by the mass of the feeding apparatus (*F*), because it affects the overall size of the animal (see Fig. S1 in Appendix S2). If the temperature range considered in comparative studies includes these critical temperatures then the effect of temperature will appear to be much greater than if it does not. Furthermore, the temperature range is predicted to interact with *F* in determining the magnitude of the body size response to temperature. For instance, for lower temperatures (< 20 °C) we see that the response of the optimal strategy to temperature is greater for large *F* than small *F* (see Fig. S1 in Appendix S2). That is, larger animals should show the temperature‐size trend more strongly than smaller animals, which is precisely what has been observed (Freckleton *et al*., 2003) but was taken to be evidence *against* the heat conservation hypothesis. We have shown that if energy requirements of a larger body are taken into account, then such observations do not rule out the importance of heat conservation in determining body size.

Interestingly, we found that the distribution of duration of interruptions to the food supply can qualitatively affect the relationship between temperature and size. To understand this, note that whatever the distribution of interruption durations, the time to use up a given level of fat shortens as temperature is decreased. Thus, a lower temperature increases the advantage of carrying more fat in terms of increased starvation resistance. However, carrying additional fat while foraging increases the energetic cost of foraging and hence increases the time spent foraging. This increases the predation risk. Thus, there are two options when temperature decreases: (1) carry extra fat and reduce starvation, or (2) reduce fat and hence reduce predation. In the short‐tail case (i.e. relatively few long foraging interruptions) carrying a little bit of extra fat has a large increase in survival resistance and this is the best option. In the long‐tailed case, with a higher proportion of long foraging interruptions, carrying a little bit of extra fat has a small effect on starvation resistance because the tail is so flat. Consequently the second option, reducing fat, is better. These effects can be clearly seen in Figure 2: when temperature decreases, starvation resistance is defended more strongly in the short‐tailed case than the long‐tailed case. In both cases the amount of muscle roughly follows the amount of fat as it is needed to maintain a low predation rate by keeping *L/M* in the region where β is small (see Fig. S2 in Appendix S2). Testing of these predictions will require data on the stochasticity of the foraging opportunity interruptions of closely related species; the contrast in size clines among shrews (Ochocinska & Taylor, 2003) offers one such possibility.

Limitations on finding food mean that animals should not always be larger as temperature decreases. Altitude may have a different relationship with body size to latitude because of the constraint of day length, which correlates with temperature along latitudinal, but not altitudinal, gradients. Along an altitude gradient, day length does not decrease as temperature decreases and so animals can respond by being larger as they can forage enough to maintain a larger body. By contrast, along a latitudinal gradient day length in winter will decline with decreasing temperature, and so increasing body size and so energy requirements is from some point no longer possible. Then further decreases in temperature could cause a decrease in body size. This is the opposite direction of the interaction as observed in one study of mammals (Rodriguez *et al*., 2008). However, we note that many mammals are nocturnal or crepuscular, and for nocturnal species foraging period will be longer in winter closer to the poles. A test of the role of day length would therefore compare the direction of this interaction for mammals and birds, as the latter are generally active only during daylight. Specifically, a data set on similar sized (nocturnal) mammals and (diurnal) birds living in the same geographical range could be used to test the prediction that the shorter days of winter constrain the responsiveness of body size in birds – but not mammals – to variation in temperature.

In the data set considered by Geist (1987) the body size of large mammals decreased with increasing latitude close to the poles, and so body size is largest at intermediate latitudes. Furthermore, no significant trend occurred near the equator. Both these trends are predicted by an interaction amongst the ecological parameters that influence size (Fig. 4c). The suggested explanation for this is net primary productivity (Geist, 1987). NPP (or eNPP, the ratio of NPP to the length of the growing season; Huston & Wolverton, 2011) as an explanation for Bergmann's rule relies on adult body size being restricted by the food supply. However, we suggest that it is food availability *per capita* that matters to growth and survival. Therefore, the eNPP rule can only be tested by including population size in the calculation of eNPP. The population size of animals is determined by births, which mostly occurs in the growing season, and death, of which a significant proportion occurs outside the growing season. Consumer population size is likely to follow the producer population size, and thus we might not expect the *per capita* eNPP to change with latitude.

Furthermore, life history theory (Roff, 2001) tells us that animals will mature at a size that trades off the benefits of size against time and predation costs, rather than as large as they can grow in a year. Therefore, it is not at all clear that animals will be as large as their food resources allow, and this is especially true for long‐lived animals such as endotherms that take several years to mature. Surviving those years – including winters where temperatures and difficulty in obtaining food – will have provided a strong selective pressure on body design. We emphasize that in determining starvation risk, it is not body size *per se* that matters, but the ratio of fuel to energy requirements. This undermines the proposed mechanism of how chronic food shortage leads to small body size (Huston & Wolverton, 2011). In any case, starvation is most likely outside the growing season, when the ratio of NPP to the duration of growing season is not relevant. Our model, based on the risks of starvation in winter, seems to be a more parsimonious explanation for non‐monotonic relationships between latitude and body size.

This focus on the growing season has led broad scale studies to concentrate on mean annual temperature (e.g. Rodriguez *et al*., 2008; Morales‐Castilla *et al*., 2012), which may be only weakly correlated with mean winter temperature. In summer, starvation is unlikely and therefore our model does not apply. Of course, there are many other selection pressures on body size (e.g. sexual selection, competition over territories), which here we have ignored, but assuming we are correct that overwinter survival influences body size evolution, then consideration of winter conditions will be required in future studies. It is especially important to quantify the food availability and the rate of interruptions to the food supply (i.e. from adverse weather) during winter. Our model suggests that the imperfect relationship between summer and winter temperatures may also help to explain mixed support for the heat conservation hypothesis.

Some studies measured skeleton dimensions to quantify body size, whereas others measure total body mass, including both lean mass and energy reserves, which may confound attempts to assign cause (Pincheira‐Donoso & Meiri, 2013). This is because an animal that is designed to resist starvation might be selected to be highly adipose, so that it has a high ratio of fuel stores to energetic requirements. As we partition lean mass from fat stores we highlight the importance of considering the type of measures of body size used. Future studies should attempt to include some estimate of relative fat, such as the size‐to‐mass ratio (or their interaction in a statistical model). To complicate matters further, adherence to Bergmann's rule may be affected by causal relationships between latitude and clutch size (Lack's rule, Lack, 1947) and between clutch size and body size (Calder's rule, Calder, 1984); and these trends may weaken one another (Boyer *et al*., 2010). We also acknowledge a problem of species richness (Meiri & Thomas, 2007) and the importance of phylogenetic inertia (Blackburn *et al*., 1999; Morales‐Castilla *et al*., 2012). In conclusion, our model does not simplify the problem of testing hypotheses about Bergmann's rule, but provides a theoretical framework that we hope will serve as a tool to reduce confusion over the mixed support Bergmann's rule has received across taxa and geographical regions.

## Biosketches

**John McNamara** is a mathematical biologist interested in the evolution of morphology and behaviour of animals. His current main interests are in evolutionary game theory, particularly negotiation strategies, and life history theory, particularly how trade‐offs are mediated by physiological state.

**Andrew Higginson** uses experimental, theoretical and comparative approaches to studying morphology and behaviour of animals. His current main focus is on integrating functional and mechanistic approaches to understanding the behaviour of animals. Other interests include investment in defences against predators in animals and plants, and specialization in cooperating groups.

**Simon Verhulst** is interested in evolutionary ecophysiology, in which function and mechanism are linked to better understand behaviour from an evolutionary perspective. His research focuses on how physiological and behavioural mechanisms mediate the trade‐offs that constitute the selection pressures that shaped behaviour over evolutionary time.

Author contributions: All authors designed the study, J.M.M. performed the analysis, A.D.H. generated the figures, all authors wrote the paper.

## Supporting information


**Appendix S1** Critical temperatures.
**Appendix S2** Supplementary figures.
**Appendix S3** Effect of temperature.
