# Supplementary material for: The influence of the starvation–predation trade‐off on the relationship between ambient temperature and body size among endotherms
Source: J Biogeogr. 2015 Dec 22;43(4):809–19. doi: 10.1111/jbi.12695 (PMC4950040; doi:10.1111/jbi.12695)
Supplement: Supplementary file 1 — Appendix S1 Critical temperatures. Appendix S2 Supplementary figures. Appendix S3 Effect of temperature. [file JBI-43-809-s001.docx]

Journal of Biogeography

**SUPPORTING INFORMATION**

The influence of the starvation-predation trade-off on the relationship between ambient temperature and body size among endotherms.

John M McNamara, Andrew D Higginson, Simon Verhulst

**APPENDIX S1. MARGINAL COSTS AT CRITICAL TEMPERATURES**

Suppose the ambient temperature is *T*. We here assume that if the mass, *M*, of the animal is such that it is above thermoneutrality then its rate of metabolic expenditure while resting is

, (A.1)

where is a constant. This is a simple approximation based on the idea that as mass increases components with different tissue specific metabolic rates scale isometrically. We also assume that

(A.2)

if the animal is below thermoneutrality. [Here the constant depends on the temperature *T*.]

The critical size at temperature *T* is the size at which these two rates of expenditure are equal; i.e. , so that

. (A.3)

Then is given by equation (A.1) when and is given by equation (A.2) when . From equation (A.1) we have

. (A.4)

From equation (A.2)

. (A.5)

Thus by equation (A.3)

. (A.6)

Comparing formulae (A.4) and (A.6) we see that as *M* increases the rate of increase of resting metabolic rate increases by a factor of at . In particular, when the marginal increase in resting energy expenditure with increasing *M* increases by 50% at *M_rest_*(*T*).**APPENDIX S2: SUPPLEMENTARY FIGURES**

**Fig. S1**: Effect of the mass of the feeding apparatus *F* (shown on lines) on the dependence of optimal lean mass *L** on ambient temperature *T*. Other parameters values as in Table 1 except *φ*=0.2, *α*=0.2, *γ*=20.

**Fig. S2**: Three functional forms and representative parameterisation for the effect of the proportion of that body that is lean mass (*L/M*) on the vulnerability to predators β: linear (dotted line); exponential (dashed line); sigmoid (solid line)

**Fig. S3:** Effect of three environmental parameters on (a) lean mass *L** and (b) energy reserves *R**: food availability *γ* (x-axis), and interruption rate *φ* and predator attack rate *α* (values shown in legend); , , *T* = –30. Other parameters as in Table 1.

**Fig. S4:** Effect of shorter day length (ρ_max_) on predictions relating ambient temperature to (a) optimal energy reserves *R*,* (b) lean mass *L*,* (c) proportion of the day spent foraging for long days (ρ_max_=0.7, solid lines) and short days (ρ_max_=0.4, dotted lines). When the limit on foraging is reached, the animal must decrease the energy demands of a larger body in order to be able to afford the greater costs of staying warm.

Fig S1


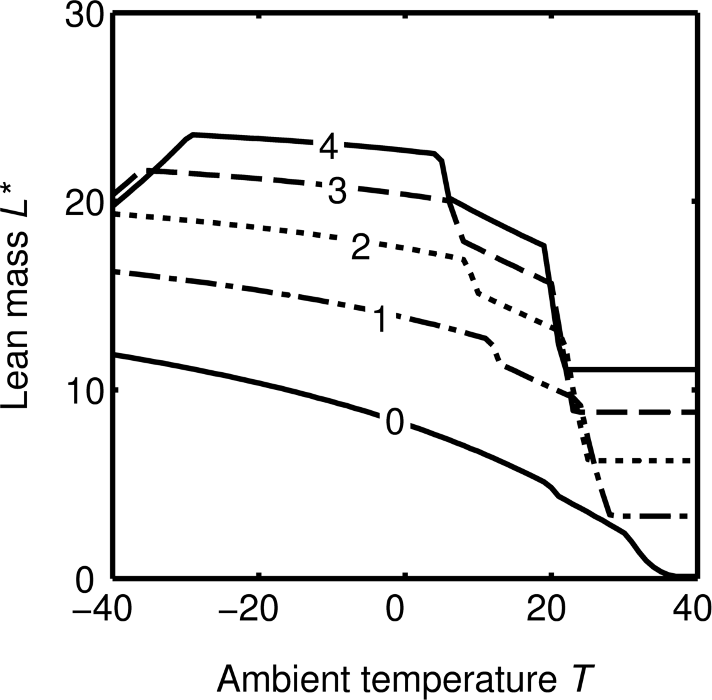


Fig S2


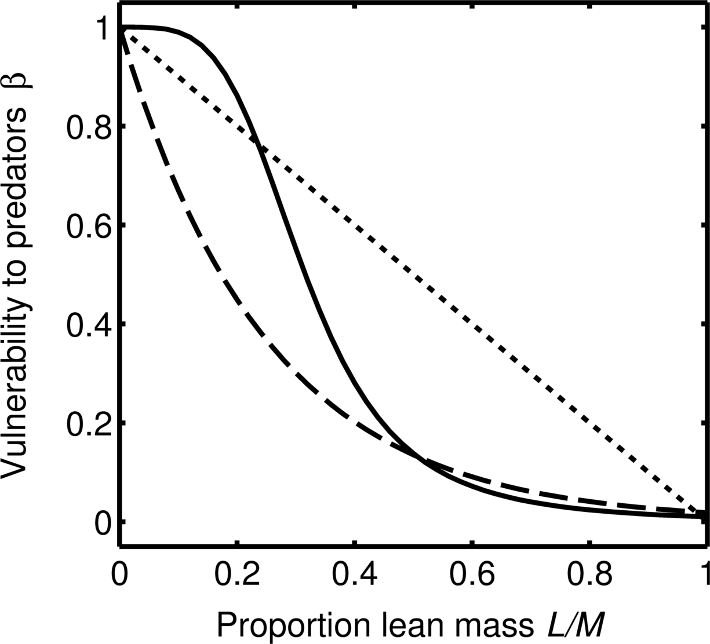


Fig S3

**
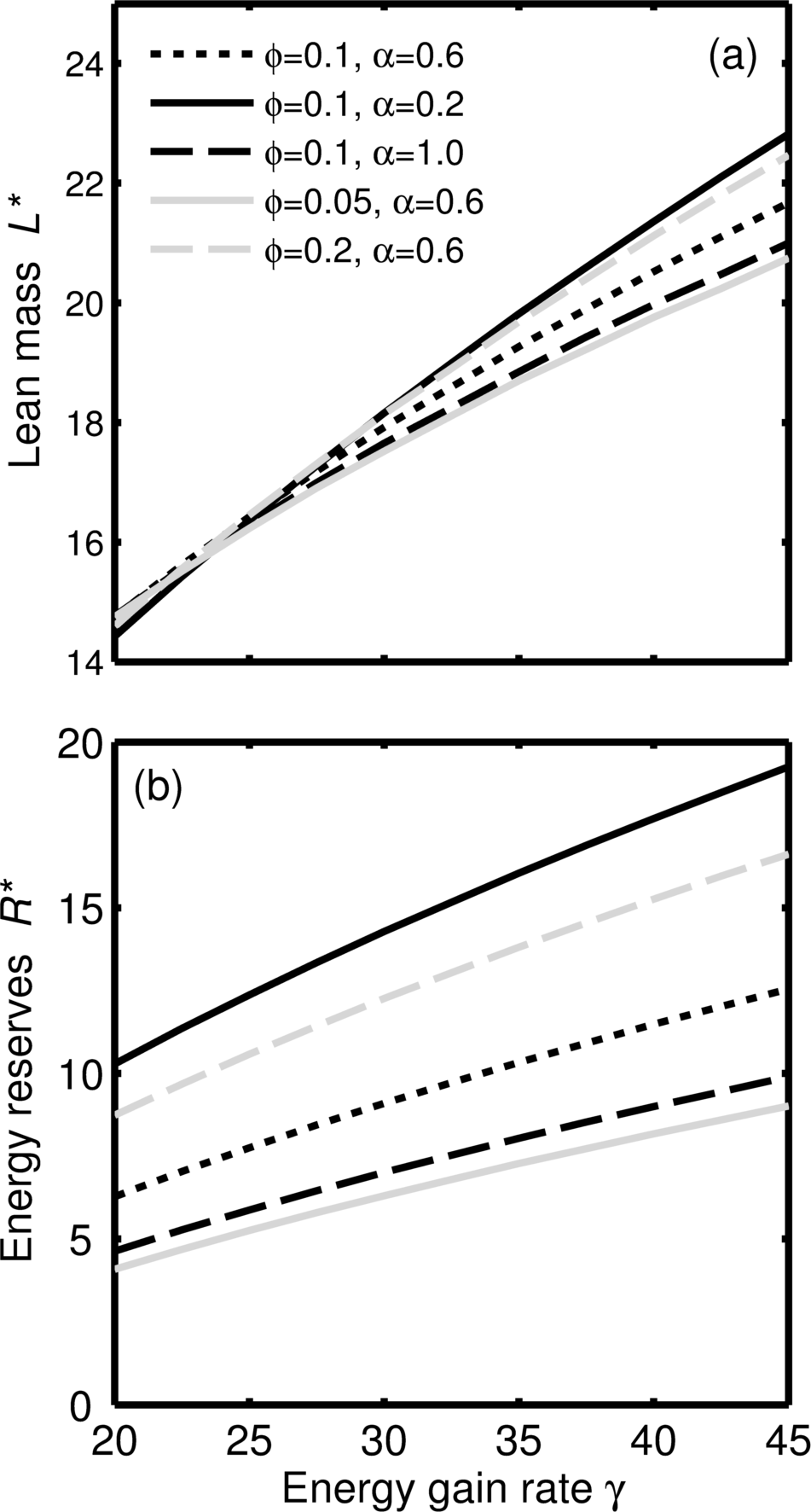
**

Fig S4

**
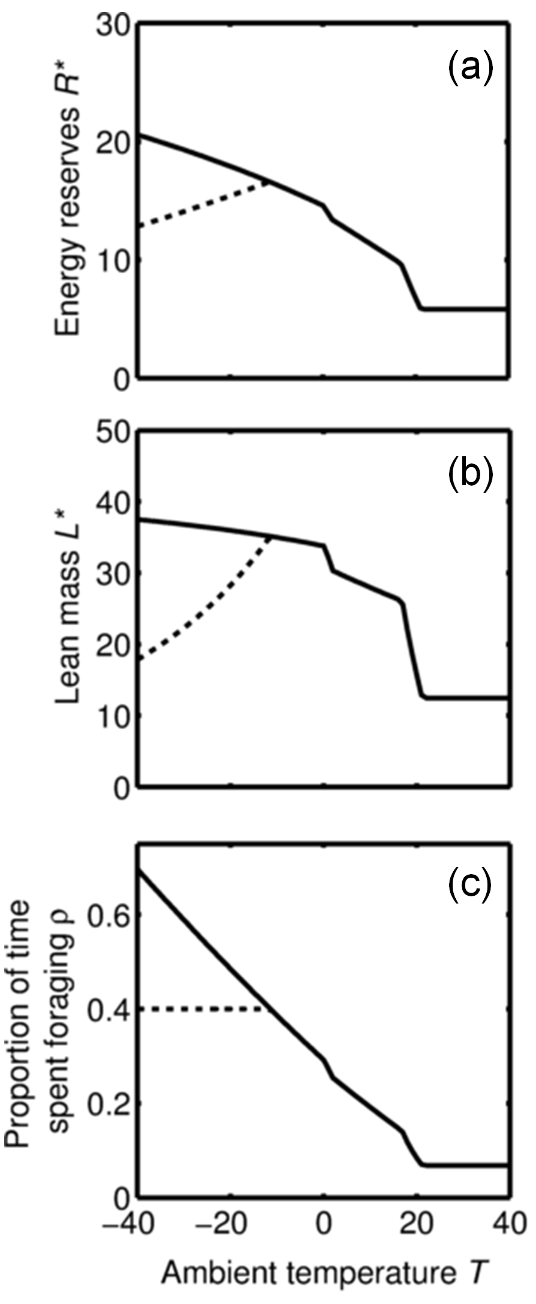
**

**APPENDIX S3: PREDICTING THE SIGN OF THE EFFECT OF TEMPERATURE**

**Effect of the distribution of interruption times under cold conditions**

Assume the temperature is so cold that even the heat generated by activity is not sufficient to keep the animal warm (), so that the rate of heat loss while resting is (equation (2)). Then the proportion of time active is

(C.1)

by equations (3) and (6). We assume that the values *L** and *R** that minimise total mortality are not extreme values, and so may be found by setting the partial derivatives of *R* equal to zero, and that at this optimum . We analyse how optimal total body mass, , depends on temperature in this special case.

Consider an animal with lean mass *L* and reserves *R*. Define

, (C.2)

and

. (C.3)

We can then express the rate of mortality *μ* given by equation (7) as

(C.4)

Expressing *γ* and $\beta$ *β* as a function of *w* and *M* rather than *L* and *R*, we can then express *μ* as a function of *w* and *M* as

(C.5)

where

$h\left( w,M \right)=\frac{{\mu M}^{\frac{2}{3}}}{I(w,M)-\left( 1-p \right)\mathrm{aM}}\beta(w,M)$ (C.6)

Assume the minimum is achieved at an interior solution (*w**, *M**). Differentiating with respect to *w* and setting the derivative to zero gives

. (C.7)

Since $h(w,M)h(w,M)$ *h(w,M)* has a minimum we also have a positive second derivative, i.e.

. (C.8)

Similarly differentiating with respect to *M* we have

(C.9)

and

. (C.10)

Both *w** and *M** are functions of temperature, or equivalently functions of θ. Taking a total derivative in equation (C.7) with respect to $\theta\theta$ gives

. (C.11)

Similarly from equation (C.9) we have

. (C.12)

From equations (C.11), (C.12) and (C.2), and setting we have

(C.13)

where

, (C.14)

and

. (C.15)

So when does *M** increase as the exterior temperature, *T* increases (i.e. the right-hand-side equation C.13 is positive)? First consider the sign of *H.* By inequalities (C.8) and (C.10) this term is the difference between two positive quantities. Its sign is therefore not clear, although its sign is positive for the examples illustrated in the figures in the main text. The sign of *J* depends on the sign of , which depends on the details of how the intake rate γ and susceptibility to predation *β* depend on body composition (see Appendix S3). Nevertheless, equation (C.13) is negative if an odd number of its terms are negative, which suggests that the sign of the function is crucial in determining whether *M** increases with temperature. To give an idea of this condition, suppose that near the function *S* is of the form where are constants, and the positive constant *b* determines the rate at which S decays*.* Then is positive if and only if $b>1b>1$. We refer to distributions for which is positive as short-tailed (in the neighbourhood of ) and distributions for which is negative as long-tailed. For the baseline cases we present in the textand , so that *M** increases with decreasing temperature in the short-tailed case and decreases in the long-tailed case.

**Comparison of different predation functions**

The signs of the functions *H* and *J* in equation (C.13) depend on the details of the values γ and *β*. It is difficult to analyse the sign of the function *H*, as this function may change sign along it range. However, when γ is constant it is relatively straightforward to analyse the sign of the function *J* in certain special cases. Let μ be given by equation (C.5). We suppose that γ is constant. Suppose also that the predation susceptibility* β* can be written as

, (D.1)

where *f* is a decreasing function. Here we investigate how the sign of the function *J* (equation (C.14)) depends on the functional form of *f*.

It is convenient to set . Since we can express *z* as a function of *w* and *M* as

. (D.2)

We consider the mixed partial derivative of the function *h* (equation (C.6)) in this special case. We can write

, (D.3)

where

. (D.4)

Note that condition (C.8) then reduces to

. (D.5)

We also have

. (D.6)

Thus evaluating this derivative at *z=z**$z=z*$ and *M=M** and using equation (D.5) we have

.

We now use the equation (D.2) and the fact that to deduce that

. (D.7)

We now apply this formula to two special cases.

***Linear predation susceptibility***

Suppose that

,

where *c* and *b* are positive constants. Then and , so that condition (D.8) becomes

.

Thus expressing the right hand side as a logarithmic derivative we can show

is an increasing function.

Thus by equation (D.4) we have

***Exponential predation susceptibility****.*

Suppose now that

where *c* and *b* are positive constants. In this case so that condition (D.8) gives

Thus, when *p*=1, if susceptibility decreases linearly with *L/M* then the function *J* is positive, while if susceptibility decreases exponentially *J* is negative.
